# Implementing tuberculosis patient cost surveys in resource-constrained settings: lessons from Tanzania

**DOI:** 10.1186/s12889-022-14607-6

**Published:** 2022-11-25

**Authors:** Andrew Martin Kilale, Charles Makasi, Melkisedeck Majaha, Chacha Dionis Manga, Sylvia Haule, Pudensiana Hilary, Omari Kimbute, Stephen Kitua, Bhavin Jani, Nyagosya Range, Bernard Ngowi, Emmanuel Nkiligi, Emmanuel Matechi, Wilbard Muhandiki, Vishnu Mahamba, Beatrice Mutayoba, Julia Ershova

**Affiliations:** 1 Muhimbili Research Centre, National Institute for Medical Research (NIMR), P.O. Box 3436, Dar Es Salaam, Tanzania; 2 Amani Research Centre, National Institute for Medical Research (NIMR), P.O. Box 81, Muheza, Tanga, Tanzania; 3 World Health Organization (WHO) – Country Office, Dar Es Salaam, Tanzania; 4 University of Dar Es Salaam, Mbeya College of Health and Allied Sciences, Mbeya, Tanzania; 5 National Tuberculosis and Leprosy Program (NTLP), Ministry of Health, P.O. Box 743, 40478 Dodoma, Tanzania; 6 KNCV Tuberculosis Foundation, P.O. Box 11013, Dar Es Salaam, Tanzania; 7 Department of Preventive Services, Ministry of Health, P.O. Box 743, Dodoma, Tanzania; 8 U.S. Centers for Disease Control and Prevention (CDC), Atlanta, USA

**Keywords:** Patient Cost Survey, Implementation, Experience, Tanzania

## Abstract

Tuberculosis (TB) disproportionally affects persons and families who are economically and socially disadvantaged. Therefore, a patient cost survey was conducted in Tanzania to evaluate the costs incurred by patients and their households before and after the diagnosis of TB. It was the first survey in Tanzania to ascertain baseline information and experience for subsequent surveys. This paper aims to share the experience encountered during the survey to ensure a standardized approach and elimination of potential barriers for the implementation of future surveys. A total of 777 TB patients from 30 clusters selected based on probability proportional to the size were interviewed during the study period. As the sample size was calculated based on notification data from the previous year, some health facilities experienced an inadequate number of TB patients during the study to meet the allocated cluster size for the survey. Most facilities had poor recording and recordkeeping in TB registers where deaths were not registered, and some patients had not been assigned district identification numbers. Fixed days for TB drug refills in health facilities affected the routine implementation of the survey as the interviews were conducted when patients visited the facility to pick up the drugs. Tablets used to collect data failed to capture the geographic location in some areas. The households of TB patients lost to follow-up and those who had died during TB treatment were not included in the survey. When planning and preparing for patient costs surveys, it is important to consider unforeseen factors which may affect planned activities and findings. During the survey in Tanzania, the identified challenges included survey logistics, communications, patient enrollment, and data management issues. To improve the quality of the findings of future surveys, it may be reasonable to revise survey procedures to include households of TB patients who were lost to follow-up and those who died during TB treatment; the households of such patients may have incurred higher direct and indirect costs than households whose patient was cured as a result of receiving TB treatment.

**Supplementary Information:**

The online version contains supplementary material available at 10.1186/s12889-022-14607-6.

## Background

Tuberculosis (TB) is a public health problem posing challenges to many families and countries worldwide [1]. The disease disproportionally affects disadvantaged persons both socially and economically. In most developing countries, TB care and treatment are provided free of charge to ensure universal access to care and reduce the economic burden on the individual and health system [2–4]. However, many TB patients and families still face high direct (medical and non-medical) and indirect costs due to TB illness and healthcare-seeking, which hampers access and puts the economically vulnerable population at higher risk of impoverishment [5–9]. These costs may be associated with longer treatment periods varying from six months for drug-sensitive TB (DS-TB) to two years for drug-resistant TB (DR-TB). Non-medical costs, such as costs for travel and food during health seeking, have been reported to be a major part of direct non-medical costs for patients, limiting access and adherence to treatment and care, which eventually affects the clinical health outcomes of patients [1, 5, 9]. By addressing barriers and reasons for the delay in initiation of TB treatment, costs incurred by TB patients can be effectively reduced [9].

Until recently, the magnitude of the economic burden incurred by TB patients and their households in Tanzania was unknown since programmatic data on costs incurred by patients while on TB care have not been collected. This information is critical for developing policies aligning with Universal Health Coverage (UHC) and aligning country efforts with global strategies to end TB by 2030 as outlined in the Sustainable Development Goals (SDGs) [10, 11]. As such, the Ministry of Health, Community Development, Gender, Elderly and Children (MOHCDGEC), in collaboration with the National Institute for Medical Research (NIMR), World Health Organization (WHO), and the U.S. Centers for Disease Control and Prevention (CDC) conducted a TB Patient Cost survey in Tanzania to evaluate the costs incurred by patients and their households before and after diagnosis of TB. The study methods and results are available elsewhere [12]. This was the first survey in Tanzania to derive baseline information and experience for subsequent surveys to be conducted regularly. The current publication aims to share the lessons learned during the implementation of the survey to inform researchers on appropriate planning and implementation of future surveys.

## Survey implementation and lessons learned

1. **Survey organization**

The first nationally representative cross-sectional facility-based TB Patient Cost survey was conducted in August–September 2019 at Tanzania's 30 facilities (clusters). The clusters were represented by facilities and selected based on probability proportional to the size of the respective facility according to the number of TB patients notified in the facility in 2017. Seven hundred seventy-seven TB patients, including children, were enrolled in the study and interviewed (no age limit was applied). All patients signed an informed consent form before the interview. In addition, parents/guardians of children under 18 years old provided informed consent for the child and responded to the interview questions. All responses were recorded using tablets in real-time in the field and uploaded to a secured web-based server for data storage. The generic electronic survey tool developed by WHO and adapted to the Tanzania context was utilized for data collection. Patients were compensated in cash for the travel and time spent during the interview. Parents or guardians of the children under 18 years who enrolled in the survey received the same compensation.

### Survey teams

The survey implementation team consisted of officers from the National TB and Leprosy Program (NTLP), national consultants and researchers from the NIMR with different professional backgrounds, and external consultants from CDC and WHO. The implementation team coordinated all survey operations. Consultants from CDC and WHO monitored the survey implementation and provided technical support to ensure quality and scientifically sound results. Five field teams with three interviewers each were formed for data collection. The composition of the field teams took into consideration individual professional qualifications to ensure there was at least one clinician and one sociologist on each team. Each field team had a leader who was a senior and experienced researcher responsible for planning the team's daily activities, solving field challenges, and maintaining cohesion among team members.

2. **Survey implementation**

Planning and execution of the TB patient cost survey is a comprehensive process and involves several steps which must be accomplished.

### Planning and organization of the survey

The availability of an implementation plan with clearly defined strategies and actions made it possible to effectively collect quality and credible data within the intended period of the survey in Tanzania. In addition, the existence of a well-functioning NTLP infrastructure (TB clinics and TB health workers) and the availability of TB treatment documentation were key to the success of the survey data collection.

### Recruitment and training of data collectors/interviewers

Appropriate selection of the interviewers was crucial to ensure effective data collection. The interviewers were recruited from the Tanzania National Institute of Medical Research (NIMR) staff. They had prior experience working in settings where the survey was conducted. After recruitment, the interviewers were trained for five days. The training focused on the study protocol, objectives, ethical issues, and data collection tools.

As a result, the experienced field team members, with the acquired skills and knowledge of their responsibilities, completed the enrollment and interviews within two months, in contrast with other similar studies [7, 13, 14] where the data collection took much longer.

### Survey pilot

Field testing of the data collection tool and patient interviews was done in three health facilities that provided TB services; refinement of the questionnaire followed the pilot exercise. The health facilities where pilot testing was conducted were not among those selected for the main survey. We learned that allocating sufficient time for training the interviewers directly influenced the quality of collected information. The electronic data collection tool required sufficient time for training and rehearsal. In addition, training and piloting provided before the survey strengthened the ability of the interviewers to use the tablets and collect information in real time.

### Availability of reliable transport

Each field team was facilitated with a well-maintained vehicle equipped with field-experienced drivers throughout the data collection period. Having a suitable vehicle and an experienced driver was an important logistical component of the successful implementation of the survey as it enabled timely visiting of the health facilities according to the field team schedule.

### Communication and coordination

Rapid and effective communication was attained through the social networking application, e.g., WhatsApp on mobile devices, facilitating data collection for the survey. Mobile phones were used to communicate with the authorities of the district and facility, especially when an immediate response was required. Any challenge experienced during data collection in the field was communicated and shared immediately to minimize carrying forward mistakes in data collection. Challenges were shared, but no patient personal information was released through the social network. Prior communication with the selected sites made the survey implementation much easier and contributed to the successful survey operations. Scheduled interviews reduced the chances of jeopardizing the quality of interviews at the TB clinic by allowing sufficient time for each interview with 2–3 interviews per day.

### The backup mechanism in case of electricity interruption

The Field teams were equipped with portable batteries, which had a sufficient storage capacity for charging the tablets and mobile phones in case the teams needed to travel long distances and work in rural areas where electricity was not available. Also, a few copies of the questionnaires in Kiswahili and English languages were printed and carried out by the interviewers for better familiarization with the questions. The paper questionnaires were used in case the tablets had technical faults during interviews. The team leaders were responsible for ensuring the team members charged their tablets and mobile phones before fieldwork.

### Survey monitoring

Survey activities were monitored through coordination and supportive supervision to ensure the implementation of the survey according to the protocol and high quality of the collected data. Each field team provided a weekly report to the Survey Coordinator to allow documentation and evaluation of the team’s performance. Daily team meetings were convened in the field to discuss the challenges encountered before uploading the data to the central server. Challenges found to be too complex and requiring further technical inputs were elevated to the Survey Coordinator. The uploaded data were monitored and checked for consistency, accuracy, completeness, and quality daily by the survey data manager in the central survey office in Dar es Salaam who generated correspondent queries and shared them with the teams. This approach provided an opportunity for the survey implementation team to review collected data and provide their feedback and technical advice to the field teams as needed.

At some points, the survey field teams served as program review teams since programmatic issues and challenges needing immediate action were raised during some of the visits. These included high death rates of TB patients after starting treatment in some districts; shortage of anti-TB medications, especially pediatric formulations; delays in updating TB registers; delays in treatment initiation; the presence of some patients who had completed a full course of anti-TB treatment yet were still kept on treatment, and others. During the implementation of the survey, health care workers were mentored by the field teams to follow the National Guidelines for TB Care and Treatment. Observed and reported deviations from guidelines were immediately reported to the district and regional TB coordinators.

### External technical assistance

Consultants from the CDC and WHO provided technical assistance during the survey implementation. Skype meetings between the in-country survey implementation team and external survey consultants were conducted regularly to evaluate the quality of the collected data. The guidance provided by subject matter experts enabled an efficient and high degree of success in implementing the survey.

3. **Challenges and lessons learned**

Some challenges, including survey logistics, patient enrollment, communications, and data management, were encountered during the implementation of the survey.

### Survey logistics and communications

Official letters to introduce the field teams to the regional and district administrative authorities were sent two weeks before the commencement of the survey. However, when field teams visited the regions and districts for data collection, it was reported that some of the letters had not been seen by the administrative offices. Arrangements for emergency officiation of the visits were done but caused substantial inconvenience for the survey teams. A similar situation caused a two-week delay in Zanzibar, where during the first site visit the field team learned approval from the Zanzibar ethical review committee was required in addition to a committee on the Tanzania mainland.

Some facilities selected for the survey did not have an adequate number of TB patients to meet the required cluster sample size requirement during the study period as the selection was based on the TB notifications from the previous year. In addition, some of the selected health facilities were not in operation at the time of the study visit, and TB patients had been transferred to other health facilities for various reasons. Thus tracking these patients was difficult. Based on our experience, it is important to contact the selected facilities before the visit to verify the status of their operations and performance and the number of patients who are registered and receiving treatment to confirm their eligibility for the survey. Also, it is urgent to plan and communicate in advance to ensure the involvement of appropriate stakeholders such as regional and district authorities and program staff; their involvement is critical for facilitating the movement of the field teams according to the survey plan and the smooth implementation of the survey.

### Patient enrollment

Each field team consisting of 3 persons was able to enroll and interview a maximum of 9 patients per day (3 patients per team member) as the survey questionnaire was long, and some questions needed additional elaboration. The interview duration (1–1.5 h) and, in some cases, long waiting time for patients to be interviewed affected the enrollment/interview procedure as most patients had limited time for attending the clinic. This resulted in pressure on interviewers and consequent errors, including transcription, typos, and even swapped information. In some circumstances, the patients were asked to come back to clarify some information given during interviews.

In some facilities, TB patients refilled their drugs once per month, especially in the continuation phase. In this situation, even in facilities with a large number of patients, it was difficult to get enough patients for interviews during the facility visits. It was also found that some of the TB clinics were opened only on fixed days in a week. Furthermore, some patients lived more than 20 km away from the health facility. To come to the facility for an interview and travel back home, these patients hired motorcycles. Waiting to be interviewed meant more travel charges, contributing to the limited availability of some patients to participate in the survey.

Our experience demonstrated enrollment of the patients for the study could be challenging and required the development of comprehensive standardized operating procedures (SOP) to ensure patients were enrolled systematically, and consistently and enrollment does not interrupt the survey operations.

In this study, we followed the WHO guidelines on TB Patient Cost surveys and did not reach the households of TB patients who were lost to follow-up or died during TB treatment [15]. However, the households with such patients could incur higher costs than households whose patients were cured after receiving the treatment. Thus, the costs due to TB and the proportion of households that experienced catastrophic costs could be underestimated. As a long-term strategy, this is an area for future research to determine if it is feasible to include in the surveys households with patients who are lost to follow up or died, and the algorithm of the inclusion of such households in the survey.

### Data collection and management

Poor recordkeeping and misplaced TB registers in health facilities were challenges during patient enrollment and potentially could affect the survey findings. During field visits, it was revealed that TB patient information in most facilities was not updated. As a result, there were records of unreported deaths and patients lost to follow-up. Some TB patients were taking their medication for more than the required period for the recommended regimen. In some areas, patients were reported to be sharing TB drugs with fellow TB patients, friends, or neighbors (those patients were not excluded from our analysis). Also, some TB patients were found to be on treatment but were not given a unique identification number as required by the program. All the above issues led to difficulties in finding their records in the unit registers.

As the survey data were collected in real-time using an electronic data collection tool, it was important to consider all the possible issues which might arise during data collection while designing the electronic questionnaire. However, the Tanzania data collection tool inadvertently omitted some restricted inputs. As a result, it was difficult to detect some errors and discrepancies between questions during interviews using the tablets. For example, some tablets were accidentally set in auto-fill mode. Therefore, if the interviewer was not careful, a different word or number might be auto-typed without notice. Additionally, due to poor internet connection, the geographic location was not captured in some areas.

For successful survey implementation, it is critical to ensure the TB registers and patient cards in the selected facilities are maintained and updated properly. The facility staff should be informed about the study before the field team’s visits to allow cleaning of TB documentation in preparation for the visits. Also, if implementing real-time data entry, ensure the data collection tool includes all possible validation codes for data entry and is equipped with a data verification module to check and clean the data in the field.

### Patient incentives

The amount of money set as patient compensation for transport appeared inadequate on some occasions. This was specifically related to patients residing far from the health facility requiring transport fares higher than the minimum amount budgeted by the survey. During our survey, some inconveniences were experienced when the transport compensation money was less than the actual transport costs for patients living very far from the selected cluster providing the patient’s care and treatment. A realistic and adequate budget is critical for the smooth execution of the survey. Therefore, appropriate budgetary considerations for patient transport costs and a modest allowance for the field support provided by health care workers from the selected facilities are important.

## Conclusions

Preparation for patient cost surveys needs to take into consideration of unforeseen factors which can affect the planned survey activities and findings. The reported successes and identified challenges during the first Tanzania TB Patient Cost Survey were documented across categories, including survey logistics, communication, patient enrollment, and data collection. This information provides insights for appropriate planning and the implementation of similar future surveys. It would be reasonable for the WHO to review the recommended guidelines for conducting TB patient surveys to improve the quality of the findings. Future surveys could include households of patients lost to follow-up and those who died. Households such as these may have incurred higher direct and indirect costs than households whose patients were cured after receiving treatment.

## Supplementary Information

**Additional file 1.** Patient cost report.

**Additional file 2.** Report Nothern Zone

**Additional file 3.** TB patient cost survey report

**Additional file 4.** TBPCS field report.

**Additional file 5.** Team number one.

## Data Availability

Not applicable.

## Abbreviations

TB: Tuberculosis
DS-TB: Drug-sensitive tuberculosis
DR-TB: Drug-resistant tuberculosis
UHC: Universal Health Coverage
SDGs: Sustainable Development Goals
MOHCDGEC: Ministry of Health, Community Development, Gender, Elderly, and Children
NIMR: National Institute of Medical Research
WHO: World Health Organization
CDC: Centers for Disease Control and Prevention
PPS: Probability proportional to size
NTLP: National TB and Leprosy Program
