# Supplementary material for: Implementing tuberculosis patient cost surveys in resource-constrained settings: lessons from Tanzania
Source: BMC Public Health. 2022 Nov 25;22:2187. doi: 10.1186/s12889-022-14607-6 (PMC9701028; doi:10.1186/s12889-022-14607-6)
Supplement: Supplementary file 1 — Additional file 1. Patient cost report. [file 12889_2022_14607_MOESM1_ESM.docx]

**DATA COLLECTION REPORT FOR TB PATIENT COST SURVEY 10/8/2019**

**Team members**

Veronique Elias; data collectors specialized in IT and mathematics.

Amani Wilfred; data collector and team statistician.

Chacha Manga; data collector and sociologist.

**Introduction**

The tasks for data collection was divided into five teams, to cover Tanzania mainland as well as island, for TB patient cost survey, in the specific selected regions and study clusters. In our case health facilities were selected to represent study clusters with the objectives of determine the economic burden incurred by TB patients and their households due to diagnosis and treatment in Tanzania. Among the three targets for the WHO end TB Strategy was to ensure that no tuberculosis (TB) patient or their household should face catastrophic total costs due to TB, and this target should be achieved by 2020. For this reason team number three (3) was assigned to collect data in the following regions; Dar es Salaam, Morogoro, Dodoma, Mbeya and Rukwa. In our case we managed to interview TB 26 in each cluster. Therefore a total of 156 TB patients were interviewed in all clusters, furthermore the exercise was executed with some minimal challenges in each study cluster.

| Serial No | Regions | Clusters visited | TB patients covered |
| --- | --- | --- | --- |
| 1 | Dar es Salaam | Infectious Disease Centre( IDC) | 26 |
|  | Morogoro | Saint Francis Designated District Hospital | 26 |
|  | Dodoma | Mpwapwa District Hospital | 26 |
|  |  | Kongwa District Hospital | 26 |
|  | Mbeya | Mbeya Regional Referal Hospital | 26 |
|  | Rukwa | Mazwi Health Centre | 26 |
|  | **TOTAL** |  | **156** |

**Dar es Salaam Region**

**At infectious diseases centers (IDC) 1^st^ to 5^th^ days July, 2019**

Team three was assigned to collect data on TB patient cost survey at infectious disease center (IDC). We arrived at facility at around 8.30 am, we reported to the facility incharge (IDC) and we explained the background of the magnitude and main objective of the study, after clarification, the incharge understood and welcome the team to begin with the study and she took us to the DOT nurses who we worked with them closely.

**Challenges on day one 1^st^ July, 2019**

The patient were very few who come to refill TB drugs because it was not a clinic day we were supposed to wait until we managed to interviews six TB patients who were in our selection criteria.

The GPS had poor reception of the satellite so is took time to pick up the coordinates.

We had discussion on some questions like no 23 and 24, then we followed the WhatsApp group discussion and clarified the matter.

**Challenges on day two, three, four and fifty days**

On these days we had fewer challenges such as for some patients when they were called through phones they refused to pick the phones and few were on safari outside Dar es Salaam.

Some fewer patients were crying during and after the interview, when they recalled back the hard way they passed to meet travelling, food and other expenses costs due to TB illness, we counselled the patients and they returned to good mood.

Some of the patient’s friends or relatives who found sent to pick drugs were not eligible to be interviewed because they had no enough information about the patient.

Some patients were in harry and were not ready for the interview.

**Morogoro region**

**At St. Francis Designated District Hospital (SFDDH) on 1^st^ - 8^th^ J ULY, 2019**

Team 3 travelled safely to Morogoro. We reported to the Regional health secretary, then he took us to RMO office where by we signed the visitors book. Thereafter the team was introduced to RAS and at last to DTLC of Ifakara town council. The next day the Ifakara DTLC introduced us to DED, DMO and the facility incharge (MOI) and we explained the background of the magnitude and main objective of the study, after clarification, the incharge understood and welcome the team to begin with the study and he took us to the DOT nurses who we worked with them closely. The same day we started the interviews.

**Challenges**

On day one; The Morogoro acting RTLC was on vacation and hence slowed down logistics. Alternatively we called him and he directed us to Regional Health Secretary for logistics assistance, who facilitated smoothly the regional protocol for responsible officials.

Some of the patients sent friends or relatives to pick drugs for them, for this reason we set appointment with the patients for next day.

Some patients were in harry and were not ready for the interview.

The GPS had poor reception of the satellite so is took time to pick up the coordinates.

**Dodoma region 8^th^ -12 July, 2019**

Team 3 travelled safely to Dodoma. We reported to the RTLC, then he took us to RMO office where by we signed the visitors book. Thereafter the team was introduced to RAS, DMO and at last the RTLC introduced the team to DTLCs of Kongwa and Mpwapwa districts because they were together at Dodoma city for trainings and reports. The same day we travel to Kongwa district whereby the Kongwa DTLC introduced us to DED and the facility incharge (MOI)of Kongwa District Hospital and we explained the background of the magnitude and main objective of the study, after clarification, the incharge understood and welcome the team to begin with the study and he took us to the DOT nurses who we worked with them closely. The same day we started the interviews.

**At Kongwa District Hospital on 15^th^ 18 and 22th July, 2019**

**Challenges**

Day one, we got few patients who came to refill TB drugs, because we started late due to the interference of logistics setting to district officials for being granted permission to begin the study.

On the followed days, we had fewer challenges such as for some patients when they were called through phones they refused to pick the phones and few were on safari outside Kongwa.

Other patients refused to come when called and this led us to wait for more than days planned to complete the sample required.

Day four the team couldn’t do any work because of president J. P. Magufuli visit that led other routine to stopped for the whole day, this made us shift to Mpwapwa district cluster for few days. And then went back to Kongwa district cluster to complete the six remained patients.

**At Mpwapwa District Hospital on 18^th^  -21^th^ July, 2019**

**Challenges**

Day one, we got few patients who came to refill TB drugs, because the activities at Kongwa stoped followed to the president visit at the district, so we decided to travel to Mpwapwa district for logistic setting that made the team to start working late at noon.

**Mbeya region 23th -31^st^ July 2019**

Team 3 travelled safely to Mbeya. We reported to the RTLC, then he took us to RMO office where by we signed the visitors book. Thereafter the team was introduced to RAS, DMO and at last the RTLC introduced the team to DTLCs of Mbeya city council. The next day DTLC introduced us to DED, then RTLC and DTLC helped the team to select the complementary cluster that met the study selection criteria because the mother-cluster had no both TB diagnostic and treatment services, for this reason Mbeya Regional Referral Hospital was found to be eligible for the study.

**Mbeya Region Referral Hospital**

**Challenges**

The selected mother-cluster did not meet the criteria of the study, the team discussed together with RTLC and DTLC on other facilities and found that Mbeya Regional Referral Hospital had all criteria for the study to be conducted while the other facility at Mbeya city council missed one criteria that was the required number of patients. The reason for that selection was because Mbeya had many DOTs and diagnostic services distributed around that caused each facility to have few number of TB patients.

On day one; we got only one patient because it was not a day for TB clinic

**Rukwa region 1^st^ -8^th^ August 2019**

Team 3 travelled safely to Rukwa. We reported to the RTLC, then he took us to RMO office where by we signed the visitors book. Thereafter the team was introduced to RAS, DMO and the RTLC introduced the team to DTLCs of Sumbawanga municipal council. The same day we were introduced to DED and the facility incharge of Mazwi health Centre and we explained the background of the magnitude and main objective of the study, after clarification, the incharge understood and welcome the team to begin with the study and he took us to the DOT nurses who we worked together.

**At Mazwi health centre in Sumbawanga**

**Challenges**

We had very few challenges compared with other health facilities such as sometimes we had to wait TB patients for long time.

We fished an exercise of data collection on 8^th^ August, 2019

**Achievements**

Apart from the challenges we experienced, we managed to finish our required sample of 156 TB patients who were eligible for all mentioned clusters and this was passible due to hardwork, professional experience and team work we had in a group three.

**Recommendations**

We recommend to confirm with regions and study sites which the introduction letters and attachments were sent to crosscheck if the authority officers had received the documents to allow smooth performance and reduce delay of process.
