# Supplementary material for: Implementing tuberculosis patient cost surveys in resource-constrained settings: lessons from Tanzania
Source: BMC Public Health. 2022 Nov 25;22:2187. doi: 10.1186/s12889-022-14607-6 (PMC9701028; doi:10.1186/s12889-022-14607-6)
Supplement: Supplementary file 2 — Additional file 2. Report Nothern Zone [file 12889_2022_14607_MOESM2_ESM.docx]

**2- REPORT NOTHERN ZONE**

**Team Members**

1. Dr Stephen J Mduma
2. Miss Purdenciana Hilary
3. Mr Ipuge Mtaka

**Introduction**

Tuberculosis patient cost assessment was conducted to assess the cost inquired by TB patients before he/she is diagnosed with TB after diagnosis and during TB treatment, house hold expenditures and assessment of working days that patients had lost due to TB illness among other variables.

This evaluation was conducted in Tanzania mainland, Zanzibar and Pemba islands in July 2019. All regions were entered in a random selection. Seventeen regions were selected and these were Dar es Salaam, Unguja, Pemba, Pwani, Morogoro Mwanza, Arusha, Kilimanjaro, Mara, Dodoma, Kagera, Manyara, Mbeya, Mtwara, Rukwa, Shinyanga and Tabora. Selected regions represented the following zones, namely Northern, Southern highlands, Eastern, Central and Lake zoned regions were selected.

A cross sectional study design was applied and study participants involved men, women and children who are TB patients and are currently on TB treatment and have completed 14 days in the course of treatment for both intensive and continuation phase.

The team collected patient information through systematic sampling of patients as they attend to the health facility to collect TB drugs. Prior arrangement was made whereby DOT nurse asked to call patients through their mobile number to insist them not to miss their TB drug pick up. Patient cluster register was used for facilities to reduce patients duplications.

**Terms of reference**

1. To conduct TB patient cost assessment following WHO guidelines
2. Conduct data analysis
3. Report disseminations

**Achievement**

The Team managed to visit all mother clusters as follows:

Dar es Salaam Kigamboni HC and Amana Hospital

Kilimanjaro Region TPC Lutheran Hospital and Njoro Dispensary

Arusha region Kirurumo Health Centre

Manyara Region Haydom Lutheran Hospital

However, there were addition complimentary clusters in Kilimanjaro and Arusha because the mother clusters could not have enough client to reach 26 for a cluster. Therefore, after discussion with coordinator it was decided to add two complimentary cluster in Kilimanjaro these were HIMO OPD and Mawenzi hospital and one cluster in Arusha region which was Mto wa Mbu HC. Himo OPD was a complementary of TPC Hospital and Mawenzi hospital was a complementary of Njoro Dispensary while in Arusha Mto wa Mbu Health Centre was a complementary cluster of Kirurumo Health Centre.

For each of 4, clusters visited in the northern zone the team managed to interview 26 clients which is 100% achievement.

Table 1: Summary of all health facilities visited and number of interviews conducted in by regions in July 2019

| **Regions/District** | **Name of Health Facility** | **Number of Interviews** | **Percent achieved** |
| --- | --- | --- | --- |
| Dar es Salaam |  |  |  |
|  | Kigamboni HC | 26 |  |
| Kilimanjaro Region |  |  |  |
| Moshi DC | TPC Hospital | 11 |  |
|  | HIMO OPD dispensary | 15 |  |
| Sub Total |  | 26 | 100 |
| Moshi MC | Njoro Dispensary | 3 |  |
|  | Mawenzi Hospital | 5 |  |
|  | Mawenzi Hospital | 10 |  |
|  | Mawenzi hospital | 8 |  |
| Sub total |  | 26 | 100 |
| Manyara Region |  |  |  |
| Mbulu District | Haydom Hospital | 26 | 100 |
| Arusha Region |  |  |  |
|  | Kirurumo HC | 12 |  |
| Monduli District | Mto wa Mbu HC | 14 |  |
| Sub Total |  | 26 | 100 |
| Over all Total |  | 130 | 100 |

**Challenges**

- The following health facilities did not have adequate number of TB patients: TPC hospital which had 14 eligible patients, Njoro dispensary which had 3 eligible Tb patients al in Kilimanjaro region.
- In Arusha region Kururumo Health center had 14 eligible clients.
- Some patient information like district number were missing in TB unit registers,
- Patient date of DX were missing for some patients
- Some patient date of initiation TB treatment compared to DX had big range especially retreatment after lost to follow up.
- Some patients sex were also missing
- Inadequate TB diagnostic facilities which made patients to travel along distance
- TB drug stock out for some health facilities e.g. Kirurumo HC.

*Note all identified issues like Missing District TB number were discussed with DOT nurse and consulted DTLC for ratification before making any corrections.

**Opportunities**

- Presence of competent DOT nurses
- Presence of competent DTLCs
- Presence of working diagnostic equipments in all health facilities visited

**Recommendations**

- NTLP to increase number of TB diagnostic facilities
- NTLP to strengthen DOT nurses through in-service up date training
- NTLP to look in to a way to support TB patients on treatment to reduce lost to follow-up
- NTLP to support community health care workers for active case finding by providing bicycles
