# Supplementary material for: Implementing tuberculosis patient cost surveys in resource-constrained settings: lessons from Tanzania
Source: BMC Public Health. 2022 Nov 25;22:2187. doi: 10.1186/s12889-022-14607-6 (PMC9701028; doi:10.1186/s12889-022-14607-6)
Supplement: Supplementary file 3 — Additional file 3. TB patient cost survey report [file 12889_2022_14607_MOESM3_ESM.doc]

**National Institute for Medical Research**

**Muhimbili Research Centre**

**
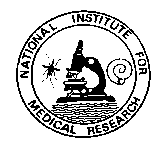
**

**SUMMARY REPORT: TB PATIENT COST SURVEY REPORT- TEAM -5**

**Members of the team**

1. Jackline Simbakalia
2. Hilda Kigola
3. Melkisedeck Majaha - Team Leader

**1.0 Introduction**

Named team was entrusted to collect data from 7 clusters to determine TB patients cost incurred in accessing health care service. Sites selected were Mwananyamala hospital from kinondoni district in Dar es salaam region replacing Emilio Health centre due to limited TB patients, others were Urambo district hospital and Nzega Hospital both from Tabora region, Rwamishenye and Katoro health centre in Kagera region and lastly Kahama hospital and Chela Health centre in Msalala district from Shinyanga region respectively.

Team managed to collect the information through interviews and obtained the required number of 26 TB patients form each 7cluster making a total of 182 attending at the TB clinic who were either in intensive phase or continuation. The information gathered will be presented according to the date of visit for each cluster in the respective region as noted below.

**2.0 DAR Es SALAAM REGION**

2.1 Mwananyamala Hospital

Data collection began on 2^nd^ of July 2019 in Mwananyamala Hospital after receiving the information from the Emilio Health centre, that they don’t have any TB patients currently attending to their health facility. Communication with study coordinator was done and the instruction was to lies with RTCL for the selection of another potential health facility which could be used to replace the initially selected facility. Consensus was achieved on selecting Mwananyamala Hospital within Kinondoni District with adequate number of TB patients. The information was sent to the DTLC of Mwananyamala Hospital, Medical officer incharge and accepted the team to start collecting the data at their hospital.

**3.0 TABORA REGION**

**3.1 URAMBO**

The team arrived at Tabora on 8^th^ July protocols were followed; after meeting with the Region TB and Leprosy coordinator (RTLC), The team failed to meet RMO as it was realized that despite of letters which were sent to the RTLC were not accepted and the RMO wanted the letters which was sent by from the Principal secretary from Local Government and Regional administration office from Dodoma whereby by the time team arrived in Tabora region found that RMO’s office had not been contact for the stated activity. The team leader managed to discuss with the RTLS concerning the introduction letters and agreed that we should contact the PI to see the possibilities of getting the letter which could be sent very soon to their office. RTLC agreed for the team to precede with the implementation of the study protocols to Urambo and informed the DTLC who introduced the team to DMO and managed to proceed with data collection after the courtesy.

On 9^th^ the team visited at the Urambo DMO office for a curtsey call. The team met acting DMO and the DOT nurse as the DTLS was on leave, after the introduction, she allowed the team to proceed with the study.

**Eligible TB clients.**

From January to July 2019, Unit register shows that had total number 214 registered TB patients, 29 losses to follow up and other clients were transferred to Kaliua district.

The interview took for 9 days to collect the data from the required sample of 26 TB patients

**3.2 Nzega District**

Team arrives in Nzega district hospital on 15^th^ July 2019 and was introduced to the DMO by TB/HIV coordinator while DTLC was attending a workshop in Tabora regional Hospital. Team had a warm welcome from the DMO of Nzega after the introduction and allowed to proceed with data collection.

**3.3 Findings:**

Nzega district Hospital has a total of 71 TB patients from January to June 2019 and eligible clients were 35 patients.

Initiatives were taken to inform the DOT provider who were real committed and participated fully in assisting the team to get the number of patients required from the clusters.

Patients Cards were sought and listed according to the eligibility criteria and those who were found to be suitable were called to come for the interview. DOT providers were informed that we will enroll all TB patients with 14 days of treatment or more for those who are still in treatment either in intensive phase of treatment or continuation phase

**4.0 KAGERA REGION**

The team arrived at Kagera on 22^nd^ of July, 2019, then visited regional hospital and met with a RMO and RTLS for the introduction. The RTLS had prior information concerning our visit which made our visit to be easy. The RMO gave the team permission to continue with the study and promised to provide any support we need. The RTLS took the team and handed to the DTLC of Bukoba municipal council to complete the logistical issues. Kagera region had two clusters namely Rwamishenye which is found in Bukoba Municipal council and Katoro Health centre in Bukoba District Council. Both selected cluster had few TB patients which could be enrolled to meet study protocol hence it was decided to find supplementary health facilities. The additional health facilities were Zamzam for the Bukoba Municipality and Izimbya Hospital which is under Faith based organization for Bukoba district which currently uses the named hospital as a designated district hospital.

**4.1 Rwamishenye Health centre.**

The team started to work at Rwamishenye health Centre after finishing the courtesy call. Team leader informed the Patron of the Health center our purposes and accepted and allowed the quests to proceed with intended job. Thereafter we requested from DOT provider to see the Unit register. Team thoroughly reviewed the Unit register and found that the facility has total number of 38 TB patients (January to July 2019) and of these patients only 13 patients were eligible, and merely 9 were accessible and interviewed others were not reachable.

- Due to limited number in Rwamishenye cluster communication were done to the Coordinator to inform about the limitation of the sampled cluster. Coordinator allowed the team to proceed with selection of the supplementary health facility and Zamzam was selected to be involved in the study.
  1. **Zamzam Health Centre.**

This health facility had a total of 42 eligible TB clients. The team managed to interview 17 clients who made a total number of 26 clients as needed.

**4.3 KATORO HEALTH CENTRE**

Team managed to pay courtesy call to the Bukoba district authority through DTLC – Dr Kelonzo, introduced the purpose to the Health secretary on behalf of the DMO who was out of office for the official duties.

DTLC informed team about the possibility of getting the required number of patients from the respective health centre. Noted for quite some time the facility had very limited patients as the TB diagnosis services relies on the Regional hospital hence limiting enrollment of patients. DTLC informed the team that currently to have only 2 TB patients under treatment.

Team together with DTLC visited the health centre and found that they have a total of 11 patients since last quarter (October – December, 2018) and had 7 patients from January – June, 2019 and only 4 patients who were eligible to participate in the study. Team leader informed study Coordinator, following the discussion, Izimbya hospital was found to be the most suitable supplementary health facility.

4.4 IZIMBYA HOSPITAL

Izimbya hospital acts as designated district hospital for Bukoba rural district. Thereafter DTLC and Team leader visited the hospital for the introduction of the study and verification of the available TB patients who could be enrolled in study. Review of the unit registers shows that they have 42 patients attending from January – June 2019; eligible clients according to our study were 28 patients.

During data collection, team received good cooperation from the Hospital workers especially those in the TB/HIV clinic. Enrollment went well and managed to recruit desired number of 26 TB patients who were either in intensive or continuation phase.

**5.0 SHINYANGA REGION**

Team arrived in the region safely, on 31^st^ July 2019 paid visit to the RMO office to finalised the courtesy call together with RTLC. Introduction of the team were done and RMO was informed about the mission to be carried out in the region. We informed the RMO, region has 2 cluster which have been sampled to participate in study which includes Kahama Hospital in Kahama District and Chela Health centre from Msalala district.

RMO allowed the team to continue to work on selected health facilities after signing guest’s book. Through RTLC informed all the DTLC from the respective selected district and appealed to ensure they provide necessary assistance to smooth accomplishment of the task in the region.

5.1 KAHAMA HOSPITAL

Team leader communicated with DTLC of Kahama who informed us that is away and has delegated the work to TB/HIV coordinator to provide all necessary support after our arrival. The Team arrived safety and taken with the TB/HIV coordinator to the DMO office and Medical officer incharge of Kahama Hospital for formalization and self introduction of the team. Thereafter the team visited TB unit for verification of the patients, understanding the clinic schedule so that planning for the enrollment could done effectively. TB/HIV coordinator narrated that normally they have daily attendance of TB patients but majority prefer to come on Mondays. The team started to work on Wednesday 31^st^ July 2019. Upon perusing the Unit Register, we noted that the facility has a total number of 408 TB patients from January to 15 July 2019 who were eligible for our study. A total number of 26 patients were interviewed as per requirements.

**5.2 CHELA HEALTH CENTRE**

Courtesy call was done to the DMO office on 2^nd^ of August 2019, and were accepted by the DTLC, who introduce the team, we used the opportunity to inform the district health authority on the purposes and we will enroll 26 patients who have taken TB treatment for the 14 or more but have not finished treatment schedules.

Thereafter visited Chela Health Centres which a little bit far about 70 Km from Kahama town, arrived and met the Incharge of the health facility and was informed about the mission and what we expect from the them. Incharge cordially welcomed the team and took them to the TB/HIV clinic unit whereby we reviewed the capacity of the health facility and the number of patients attending at TB units and being registered for the effective enrollment of the patients.

Unit register showed that they had about 56 patients who are enrolled and among them 31 patients were eligible to participate in the study. January to March had 33 TB patients mean while they have 16 patients from April to July 15^th^, 2019, thus had a total of 49 patients.

While enrolling patients for the study we have found that more than 10 patients did not have district identification number (ID) whereby later we had to inform DTLC and allocated new district registration numbers, all of them were enrolled these patients were enrolled and had a range of period since were diagnosed was between (March - early July 2019). The enrollment went slow due to majority of the patients were living in remote areas and the cost to come to the health facility by motorcycle was ranging from 10 – 20,000Tsh round trip which could not certainly afford to come while their date for replenishment of the anti TB drugs was not reached.

**6.0 Achievement**

Team managed to collect a total of 182 of TB patients attending at the TB clinic who were either in intensive phase or continuation from 7 clusters.

**Challenges:**

1. Majority of the patients attending in Mwananyamala hospital are drug addict and some of them are on treatment with Methadone. Adherence to treatment noted to pose great difficulties especially in making follow up and many don’t have reliable address which can be used to track them. Also number of MDR-TB is high among those observed which could be attributable to interrupted treatment.

Basing on these observation majority don’t have reliable sources of income to assists them in incurring cost for food and travelling to access treatment properly also provides wrong physical address and mobile numbers which complicate tracking of the patients progress/follow up.

1. Some TB patients have written wrong contact address including Mobile telephone numbers
2. Many TB patients were staying in some remote areas where the network is not well connected; it took more than three days for the patients to get sms for the invitation (Urambo, Nzega, Chela)
3. Lost to follow up seems to be a big challenge as Community Health Care workers focuses on HIV clients
4. Laziness and carelessness on TB clinic staff, DOT nurse dot know the status of their clients e.g on our arrival we came to realize that 9 patients passed away long time but they were not identified and had no any idea about their attendance and adherence to treatment. Also could not know the expected number of clients who are supposed to come on a particular day of the clinic.
5. Lack of knowledge to TB patients posed more difficulties as some TB clients believe that they have witched therefore they came to the health facility very late/ on the critical stage.
6. Some patients had missing information in their cards including age, date of diagnosis and initiation of treatment and some had missing information of their HIV status
7. Some patients had no current ID in their Unit register despites of fact some have started treatment since February 2019.
8. Status of treatment was missing in the unit register which made difficult to ascertain patients whether have cured, completed treatment or still undertaking treatment
9. Patients were coming from remote areas and had problem in turning up for treatment regularly
10. Some of the health workers wrongly informed patients who were diagnosed to be TB cases with (PTB+) to continue using other antibiotics and delaying timely treatment eg patients with registration 1401/KK/19/224 who was diagnosed on 5^th^ of May 2019 but started treatment on 10^th^ June 2019. All effort were taken to ascertain the observed discrepancy by doing verification from Laboratory register, unit register and patients card it was confirmed that dates were correct. Patients’ interview revealed that some of the health workers advised the patients to take other medicine until the condition was deteriorating and thereby patients decided to report to the TB unit and start Ant TB therapy.
11. Patients had only two named which sometimes complicate the follow up of the patients outcome
12. Some had continuously treated as relapse with 5^th^ episode concurrently
13. Some patients have died and the information was not updated and found during the attempt of calling them to come for the study, 10 patients were found in Urambo hospital.
14. Majority of patients were diagnosed through X ray as compared to smear microscopy and Gene Xperty.
15. Three patients were found to have more than 3 episodes and only one detected to be MDR patients under treatment in Izimbya Hospital and one patient had 5 episodes still on 1^st^ line regimen of TB in ZamZam health centre which probably need more exploration to find other possibilities apart from TB.
16. Displacement of Unit register was more common to some of the health facilities.
17. Some of patients died since January 2019 but still they were recorded to continue taking Ant TB to August 2019.
18. Service provision in Chela health centre was normally delayed up to 10am while waiting for the staff to clean and begin the routine work.
19. Majority of TB patients were those who come late to the health facility unlike to many other clinics which we have managed to pass through while collecting data for this study.
20. Improper TB cases management. There was a patient who started treatment at a private hospital. She was ordered to have a TB test at a government hospital. Results showed that she was AFB positive. When returning those results at the private hospital, they disregard it as TB and went on operating the patient claiming that what she was suffering is tonsillitis. The patient continues to suffer after that operation until when she decided to go to a government hospital for the test again, where she was found to still have TB and started treatment. It is at this time of our survey we got to meet her and know her story.
21. On 6 &7^th^ of August Car broke down and the team could not travel to Chela as well as on 8^th^ and 12^th^ of August as being public holiday, hence interfered with team planning. During car breakdown team has to work at Kahama hospital and assist each other in finishing data collection in that cluster.

**Comments**

1. Close supervision from RTLC should be enhanced despite of the current good work as it was also noted that DTLC could not differentiate between time period for classifying and individual when is cured, completed and relapse. This has been noted to have implication in repeated treatment without further exploration of the other causes apart from TB.
2. More collaborative on job supervision between TB and HIV program should be strengthened to quality and timely update of data. Linking the missing data and Etl which does not allow update this could be contributing low notification of the majority of patients.
3. The involvement of the community health workers should be strengthen for easy tracking of the TB patients as it was experienced in Chela Health centre in Msalala district as majority of patients are livestock keepers and live distant from the Health facility.
4. Investing in training of the Community health workers could be best alternative through creation of awareness on the important of patient’s adherence treatment and improving the quality care.

**Recommendation:**

More emphasis should on improving the supervision to promote timely diagnosis and probably strengthen use of the Microscope and Gene X perty for the diagnosis of the TB cases rather than use of the radiology and clinical acumen as it has been observed some patient which routine kept on Ant TB treatment up to 5 episodes.

The exercise was officially closed on 15^th^ August 2019 after obtaining the required sample size.

I hereby submit the report on behalf of team- 5

**Dr Melkisedeck L Majaha- Team Leader**
