# Supplementary material for: Implementing tuberculosis patient cost surveys in resource-constrained settings: lessons from Tanzania
Source: BMC Public Health. 2022 Nov 25;22:2187. doi: 10.1186/s12889-022-14607-6 (PMC9701028; doi:10.1186/s12889-022-14607-6)
Supplement: Supplementary file 4 — Additional file 4. TBPCS field report. [file 12889_2022_14607_MOESM4_ESM.docx]

**FIELD REPORT**

**TASK:** Data collection for TB Patient Cost survey (TPCS)**.**

**SITES:** Five Clusters in Mwanza and Mara regions.

**DURATION:** 35 days

This work was carried by team #4 which was comprised of the following people,

1. Wilbard Muhandiki-Research assistant
2. Getrude Richard-Research assistant
3. Omari Kimbute-Research assistant and team lead
4. Frank Mutagabwa-Driver

Before commencing of the work at Lake Zone the team was involved in data collection from TB patients being attended at Tambukareli Dispensary in Temeke District, Dar es Salaam region from 1^st^ July to 5^th^ July 2019. This was considered as continuation of training and data collected was uploaded online while the supporting survey documents such as consents and registers were submitted to the coordinator before the team started off to Mwanza.

Dr. Benard Ngowi made a supervision visit during the survey. He visited the team while at Mwanza from 14/07 to 19/07/2019 and observed data collection, conducted debrief meetings after the end of each working day. All administrative/technical issues were discussed and those which required consultation with the coordinator was directed to him.

**MWANZA REGION:**

The team arrived at Mwanza on 7^th^ July 2019 and the next day paid a courtesy visit to the regional authorities including the RAS and RMO under the guidance of the acting Regional Tuberculosis and Leprosy Coordinator.

On the same day the team visited the sampled clusters from Mwanza South District and these were Bugando Zonal referral hospital and Mwananchi private hospital. The initial observation was that for Mwananchi cluster there were only three patients taking drugs from this hospital. For Bugando there were enough patient and we were ascertained of getting 26 patients as per protocol.

Consultation was made with the survey coordinator to solicit another cluster to complement for Mwananchi hospital and Nyamagana District hospital was identified. The team shifted to Magu on the evening of 16^th^ during and for two days worked at Magu hospital, a third cluster in Mwanza region. Data collection for Mwanza region was conducted as shown in the table 1.

Table 1.Data collection for Mwanza clusters.

| **Date** | **Health Facilities** | | | |
| --- | --- | --- | --- | --- |
|  | **Bugando Hospital** | **Mwananchi Hospital** | **Nyamagana hospital** | **Magu hospital** |
| 09/07/2019 | 12 | 01 |  |  |
| 10/07/2019 | 07 | 02 |  |  |
| 11/07/2019 | 04 |  |  |  |
| 12/07/2019 |  |  | 10 |  |
| 15/07/2019 |  |  | 11 |  |
| 16/07/2019 | 03 |  | 2 |  |
| 17/07/2019 |  |  |  | 14 |
| 18/07/2019 |  |  |  | 12 |
| **Total** | **26** | **03** | **23** | **26** |

**MARA REGION:**

The team moved to Mara region on 21^st^ July 2019 and on 22^nd^ made a courtesy call to the RMO and RAS offices. All the DTLC were attending a training at Musoma and we utilized this opportunity to discuss on the sites we were intending to visit. Earlier communication with RTLC showed that there were enough patients at Manyanyama health centre while Butiama District hospital regardless of being main hospital for the District the eligible patients were less than 12. The team consulted the survey coordinator and Buhemba dispensary (initially we were told that it was a health centre but in actual fact it is an improved dispensary) was selected to cover for the deficit.

Special consideration should be taken for Buhemba area where the team interviewed five MDR patients taking medication while one was waiting for MDR drugs to be brought. The area is known for gold mining and although the mine is closed but the population is still high. Data collection for Mara region was conducted from 23^rd^ to 29^th^ July as shown in the table 2.

Table 2: Data collection for Mara clusters

| **Date** |  |  |  |
| --- | --- | --- | --- |
|  | **Manyamanyama Health centre** | **Butiama district Hospital** | **Buhemba dispensary** |
| 23/7/2019 | 14 |  |  |
| 24/07/2019 | 12 |  |  |
| 25/07/2019 |  | 8 |  |
| 26/07/2019 |  | 2 | 14 |
| 29/07/2019 |  |  | 2 |
| **Total** | 26 | 10 | 16 |

On 30^th^ July 2019 the team travelled back to Mwanza and worked on data queries as reported by the data manager and also made several phone contacts with clusters for clarifications on the observed shortcomings. For queries that originated from the facilities within Mwanza town, team made a physical visit to the sites and clarified the inconsistencies with the concerned DOT nurse.

Team started off to Dar on 2^nd^ July for report writing and returning of survey tools.

**POSITIVES:**

1. Transport-The team was privileged to have a well-maintained 4x4 vehicle that did not breakdown throughout the duration of the survey.
2. The driver was punctual and well behaved young man.
3. Remuneration: The payment to research assistants and health facility workers was made on time. The allowance of 15000/-Tshs to each participating patient as transport reimbursement was well appreciated. There was a mix-up for payment to Mwananchi and Nyamagana health care workers but this was discussed and it was agreed that the payments will be made in the week ending 2^nd^ of July 2019.
4. Prior communication with the sites-This made life much easier for the team and beside Mwanza, the other places the team went the preparation was superb and team was able to capture the patients who were already at the facilities by 7.30 am. This helped the team to interview more patients without jeopardizing the quality of work as there was enough time.

**CHALLENGES:**

LOGISTICS: The agreement between the car owner and the team was not transparent and in some occasions the driver complained of delay of funds for fuel. Although this did not affect the work but it delayed the team on the day of leaving Dar es Salaam and we have to spend a night at Igunga. Hopefully, if we happen to conduct another survey the running condition of the vehicle should be well known to the team members.

PROTOCOL-sampling process allowed for inclusion of clusters with very few patients. This necessitated to look for another clusters that in turn had more patients than the mother cluster.

QUESTIONNAIRE: There were few instances where elaboration was sought because of some shortcomings. There were other few issues like,

- Although the questionnaire allowed for guardian to be interviewed but the questions were directed to the patients.
- In some questions the checking system was not in place and this allowed for RA to proceed even for occasions where this could not happen e.g. Insurance question.
- In the column for the total amount spent by the patients for consultation, treatment, laboratory and radiology maximum figure which can be accommodated is 1,000,000/=Tshs which make it difficult to put an amount higher than 1,000,000/=Tshs.

The team express its sincere thanks to the survey coordinator, WHO through Dr. Bharvin, the Supervisor, Dr. Ngowi and all those who made prompt responses to issues raised during field work.
