# Supplementary material for: Implementing tuberculosis patient cost surveys in resource-constrained settings: lessons from Tanzania
Source: BMC Public Health. 2022 Nov 25;22:2187. doi: 10.1186/s12889-022-14607-6 (PMC9701028; doi:10.1186/s12889-022-14607-6)
Supplement: Supplementary file 5 — Additional file 5. Team number one. [file 12889_2022_14607_MOESM5_ESM.docx]

**TEAM NUMBER ONE PTB-COST SURVEY**

**FIELD REPORT**

**Composition of the team**

1. Dr, Charles Makasi (Team Leader)
2. Mrs Sylvia Audi Haule
3. Mr. Bernard Katende

**Introduction**

The aim of this research study was to assess the costs (both direct and indirect) associated with a diagnosis of TB and care and treatment. The study was restricted to persons who have started TB treatment. Team one was given a total of 5 clusters as listed below;

1. ST. Benedicts Health Centre (Dar es salaam)
2. Nanyanga Dispensary (Tandahimba)
3. Lugoba Health Centre (Chalinze)
4. Mnazi mmoja National Hospital (Zanzibar)
5. Micheweni Cottage Hospital (Pemba)

At each given cluster; a minimum of 26 participants were included in the interviews. From the selected health facilities Clusters), patients who were on TB treatment when visiting the facility were systematically enrolled. Consecutive patients attending the facility were invited for interviews. In the study we intended to enroll equal number of patient in intensive and continuation phases especially at the facilities which could have adequate number of patients. This was planned to be maintained to obtain a good balance between the two groups and ensure a sufficient number of people reporting pre-treatment cost data. In case of the facilities with small number of patient during the survey period; an alternative way was planned whereby alternatively it was intended to interview all patients regardless of the phase of treatment.

Individuals who were on TB treatment were included in this study including children <18 years old. Patients on MDR-TB treatment were also included, being at public or private facilities within the NTLP network. Patients with less than 2 weeks of anti-tuberculosis treatment were excluded.

**Achievements and challenges**

Generally all required number of patients per each cluster was available; although almost all of the clusters didn’t have adequate patients and were complimented by other facilities (except Mnazi-mmoja Hospital- Zanzibar).

The research team was formed of committed and experienced personnel from different background and this was the secret towards strength of this team.

Otherwise induvial achievement and challenges are shown in the table 1 below;

**Table1. Achievement and challenges of individual clusters**

| **Cluster name** | **Achievement** | **challenges** |
| --- | --- | --- |
| ST. Benedict’s Health Centre (Ilala-DSM) from the 1^st^ to 5^th^ July 2019. | - Managed to get adequate number of patients of required sample size. (26 patients) | The mother Cluster (ST. Benedicts Health Centre was found to have only one patient. This patient not only transferred from another facility, but was not living around the facility neighbourhood. It took three days to meet the patient due to difficult communication. The rest of the patients from this cluster were obtained from complimentary facility which was Buguruni Health Centre. |
| Nanyanga Dispensary (Tandahimba- Mtwara) from the 7^th^ to 12^th^ July 2019 | - Adequate sample size (27 patients) - Good collaboration with TB staff | - Nine out of 11 eligible patients at this dispensary were available for interview - Facilities with similar or closer to similar levels were found to have very low number of patients on treatment. Therefore after discussion with coordinators; Tandahimba District Hospital was opted to avoid having more than one complimentary facilities |
| Lugoba Health Centre (Chalinze- Pwani) from 14^th^ to 22^nd^ July 2019 | - Adequate number of patients obtained (29 patients) | - Eight out of 9 eligible patients at Lugoba HC were interviewed here. A lot of efforts were used to get the remaining one patient without and success. - Complimentary facility (Ubena Prison dispensary was used to complete the required sample size - Most of patients at Ubena Prison Dispensary are pastoralists and their availability was hard due to lack of mobile communication and dynamic character of their community. |
| Mnazi mmoja National Referral Hospital, Zanzibar (Mjini Magharibi-Urban-west ) from 6^th^ August- 12^th^ August 2019 | Adequate number of required sample size. (26 Patients)   - All of 26 patients were obtained from a single facility. | - It took about two weeks to obtained research permit to work in Zanzibar - Researchers stayed in different areas due to inadequate number of Lodges in Zanzibar. Transport was also very difficult for researchers to the workplace and moving around - One researcher fell sick for about three days and hence taking care of the researcher at the same time making sure the task is performed well was a challenge. |
| Micheweni Cottage Hospital, Pemba from 13^th^ to 20^th^ August, 2019 | Obtained a required number of patients | Micheweni cottage hospital which was a mother cluster was found to have only 5 patients in the register. Of these five patients, only two were available for interview. Three of them died (Came late for treatment) details of their death were taken.  Therefore Wete Hospital was used as a complimentary facility; but again, most of the patients didn’t have contact numbers. There is no specific day for clinic and therefore patients attend in unexpected day and numbers every day. |

**General challenges**

Balancing of the patients due to their phase i.e. equal number of patients in both intensive phase and continuation phase was not well practiced due to inadequate number of eligible patients in most of the cluster facilities.

Some of the patients had their information missing from their records. Missing information appeared frequently on District TB registration numbers, contact addresses, gender, HIV status and type of TB.

The problem of missing information was resolved in most of records. This was done in collaboration with TB clinic staff and patients themselves once they were available.

**Recommendation(s)**

In the future; if there will be any study which is also supposed to be conducted in Zanzibar, process for obtaining research permit from Zanzibar should start early.

Before starting of any field activity that involves public institutions, we should go through the calendars and select the time period which contains the minimal number of public holidays.

…………………………….………END OF REPORT……………………………………….
